# Microvesicle-Derived Redox Signatures as Mediators of Endothelial Dysfunction in Diabetes

**DOI:** 10.3390/ijms27136005

**Published:** 2026-07-04

**Authors:** Sarah Khalaf Ghanem, Hanan H. Abunada, Shahenda Salah Abdelsalam, Loulia Bader, Abdelali Agouni

**Affiliations:** 1 Department of Pharmaceutical Sciences, College of Pharmacy, QU Health, Qatar University, Doha P.O. Box 2713, Qatar; sarah.ghanem@qu.edu.qa (S.K.G.); lbader@qu.edu.qa (L.B.); 2 Biomedical Research Center, QU Health, Qatar University, Doha P.O. Box 2713, Qatar; habunada@qu.edu.qa (H.H.A.); sabdelsalam@qu.edu.qa (S.S.A.)

**Keywords:** extracellular vesicles, diabetes, endothelial cells, redox, oxidative stress, diabetic complications

## Abstract

Chronic hyperglycemia and excessive reactive oxygen species (ROS) production are defining features of endothelial dysfunction, a key driver of diabetic vascular complications such as diabetic nephropathy. Microvesicles (MV-enriched fraction), a subtype of extracellular vesicles, and the stress-responsive antioxidant protein Sestrin2 (SESN2) have emerged as important contributors to these processes. This study investigated the role of the MV-enriched fraction in endothelial cell communication under diabetic conditions, with a particular focus on oxidative stress signaling. To model diabetic injury, EA.hy926 endothelial cells were treated with methylglyoxal (MGO), and the resulting MV-enriched fraction was isolated and then applied to two recipient models: naïve endothelial cells and SESN2 knockdown (KD) cells. Protein expression of key antioxidant markers, including endothelial nitric oxide synthase (eNOS), was assessed by Western blot. Nitric oxide (NO) bioavailability was quantified via nitrite measurement using 2,3-diaminonaphthalene (DAN), while mitochondrial and cytosolic ROS levels were evaluated using MitoSOX and dihydroethidium (DHE), respectively. Results demonstrated that the MV-enriched fraction derived from diabetic conditions triggers a complex antioxidant response in healthy endothelial cells, characterized by upregulation of SESN2, superoxide dismutase 1 (SOD1), and heme oxygenase-1 (HO-1). This suggests a compensatory mechanism that mitigates oxidative stress. Notably, SESN2 KD cells exhibited increased ROS production and reduced NO levels upon MV treatment, underscoring the essential role of SESN2 in maintaining redox homeostasis. Overall, this study highlights the dual role of the MV-enriched fraction as a mediator of both protective and detrimental redox signaling in diabetic endothelial dysfunction and suggests potential therapeutic targets for managing diabetic vascular complications.

## 1. Introduction

Diabetes mellitus poses a significant risk to global health, with its related complications becoming a prominent public health concern. Type 2 diabetes mellitus (T2DM) constitutes about 90% of global diabetes cases [1]. Vascular complications of diabetes are the main culprits in diabetes-related morbidity; these include CVDs, cerebrovascular disorders, nephropathy, retinopathy, and peripheral vascular diseases. This notion underscores the need for proper management of these conditions. Endothelial dysfunction is a pivotal factor in the onset of various vascular complications associated with T2DM. This process involves complex interactions among molecular processes, including oxidative stress, inflammation, and multiple cellular disruptions [2]. However, despite extensive investigation, the exact molecular determinants underpinning these pathways remain inadequately understood.

A healthy endothelium plays a crucial role in maintaining vascular health by balancing various opposing processes. These include vasodilation and vasoconstriction, growth inhibition and promotion, antithrombotic and prothrombotic activities, anti-inflammatory and pro-inflammatory responses, and antioxidant and pro-oxidant effects. When this delicate balance is disrupted, endothelial dysfunction occurs [3,4]. One of the most studied implications of the endothelium in human disease and its related complications is the balance, or rather the imbalance, between nitric oxide (NO) and reactive oxygen species (ROS) [5]. This is termed “redox balance” or “redox signaling”. Redox signaling pathways play a crucial role in regulating endothelial function. In healthy endothelial cells, a delicate balance exists between sources of oxidative stress and antioxidant systems. Perturbation of this homeostatic balance leads to a cascade of events, most prominently a reduction in NO bioavailability, a key molecule in vascular health, by either decreasing its production or increasing its degradation. Another consequence is the activation of pro-inflammatory pathways, which alter vascular tone and promote atherosclerosis. Several sources contribute to ROS production in endothelial cells, including nicotinamide adenine dinucleotide phosphate (NADPH) oxidases, mitochondrial electron transport chain, and uncoupled endothelial NO synthase (eNOS). The interplay between these ROS-generating systems and antioxidant defenses determines the redox state of endothelial cells [6].

The cellular antioxidant defense system, especially within endothelial cells, is extensive and can be categorized into enzymatic and non-enzymatic components. Enzymatic antioxidants include sestrin2 (SESN2), superoxide dismutases (SODs), catalase, and heme oxygenase-1 (HO-1), while non-enzymatic antioxidants include glutathione, vitamins C and E, and uric acid [7,8,9]. SESN2 is a stress-inducible antioxidant protein of the family sestrins (SESN1, SESN2, and SESN3), which is upregulated in cells under stressful situations, including oxidative stress [9,10]. SESN2 has been shown to mitigate endothelial dysfunction, and its implications have been associated with different pathologies such as cardiovascular disease (CVD) and diabetes mellitus [11,12,13,14,15].

Over the past two decades, extracellular vesicles (EVs) have emerged as crucial players in the pathophysiology of endothelial dysfunction in diabetes, offering new insights into potential treatment targets [13,16]. EVs serve multiple cellular functions, including the dissemination of signaling molecules, stress response, and cellular waste management [17,18]. The release and content of EVs are significantly influenced by oxidative stress, a complex cellular condition. These vesicles can carry diverse cargos, including oxidized lipids, microRNAs, and proteins, which may have detrimental effects on recipient cells. However, EVs released under oxidative stress conditions also transport antioxidant molecules, potentially protecting target cells from further damage by modulating their oxidative stress response. This dual nature of EV cargo makes it a valuable tool for understanding the progression of oxidative stress-related pathologies. The molecular composition of these stress-induced EVs can provide crucial insights into their role in disease pathogenesis and development. Furthermore, understanding the molecular mechanisms governing EV release is essential for devising innovative therapeutic strategies. These approaches could focus on either eliminating harmful components from EVs or enhancing their capacity to deliver antioxidants, ultimately addressing oxidative stress-related pathologies [19].

Despite the well-established role of microvesicles (MV-enriched fraction), a subset of EVs directly shed from the endoplasmic membrane of apoptotic or activated cells, in mediating diabetic vascular complications [17], the mechanisms by which the MV-enriched fraction mediates redox regulation and the involvement of stress-responsive antioxidant proteins such as SESN2 remain incompletely understood. Notably, previous studies investigating the MV-enriched fraction in diabetic vascular injury have largely focused on its capacity to enhance reactive oxygen species (ROS) production, with limited attention to the regulatory mechanisms that may modulate this response, including antioxidant defense pathways and endothelial nitric oxide synthase (eNOS) signaling. Based on this gap in the literature, we hypothesized that the MV-enriched fraction generated under diabetic conditions may also convey cytoprotective signals to naïve endothelial cells, activating compensatory antioxidant responses that limit the propagation of oxidative stress and attenuate methylglyoxal (MGO)-induced endothelial injury. Accordingly, this study aimed to elucidate the relationship between SESN2 and MV-mediated signaling in diabetes-associated endothelial dysfunction, a central driver of diabetic vascular complications. Defining these pathways may uncover novel therapeutic targets to preserve endothelial function and mitigate the progression of diabetic vascular disease.

## 2. Results

### 2.1. Methylglyoxal-Induced Endothelial-Derived MV-Enriched Fraction Elevates ROS Generation in Naïve Endothelial Cells

**Figure 1:**
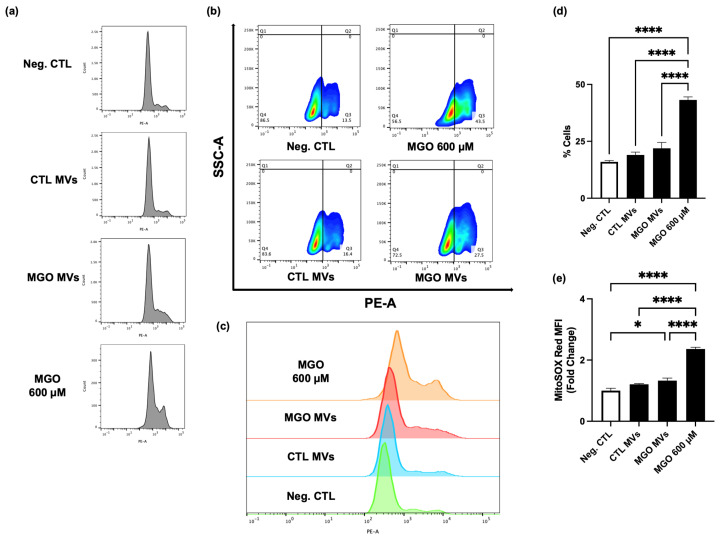
Mitochondrial Superoxide Analysis by Flow Cytometry Using MitoSOX Red in Live EA.hy926 Cells. Flow cytometry analysis of mitochondrial superoxide levels in live EA.hy926 cells using MitoSOX Red. Cells were left untreated, treated with control MV-enriched fraction or MGO-induced MV-enriched fraction (20 µg/mL, 24 h), or incubated with MGO (positive control; 600 µM, 18 h). (**a**) Representative histograms of MitoSOX Red fluorescence at 24 h. The Y-axis represents the number of cells emitting fluorescence in the PE spectral region, while the X-axis represents the average fluorescence intensity. (**b**) Representative pseudocolor plots illustrating fluorescence distribution across experimental groups; the X-axis represents MitoSOX Red fluorescence detected in the PE channel, and the Y-axis represents side scatter area (SSC-A). (**c**) Representative half-offset histograms comparing fluorescence intensity distributions among all groups. (**d**) Bar graph showing the percentage of MitoSOX Red-positive cells (mean ± SEM) for each condition. (**e**) Bar graph depicting the fold change in median fluorescence intensity (MFI) (mean ± SEM) across different treatments. All analyses were performed in at least three independent biological replicates; where each replicate represents independently treated endothelial cell culture wells/monolayers. * *p* < 0.05, and **** *p* < 0.0001 vs. Neg. CTL or indicated groups.

MGO is a highly reactive dicarbonyl metabolite produced by glycolysis that serves as an AGE precursor formed by the fragmentation of triose phosphates under hyperglycemic conditions, with subsequent activation of the receptor of AGEs (RAGE) in endothelial cells [20]. Exposure of naïve endothelial cells to the MV-enriched fraction derived from MGO-treated endothelial cells induced a pronounced increase in ROS production, notably at the mitochondrial level (mitROS), as measured by MitoSOX Red-based flow cytometry (Figure 1). Flow cytometry histograms and pseudocolor plots (Figure 1a,b) outline the gating and fluorescence profiles for four experimental conditions: untreated negative control (Neg. CTL), control MV-enriched fraction (CTL MV-enriched fraction), MV-enriched fraction from MGO-challenged cells (MGO MV-enriched fraction), and direct MGO exposure (600 μM). Overlaid histograms (Figure 1c) show a rightward fluorescence shift in the MGO MV-enriched fraction and MGO groups, indicating elevated mitochondrial superoxide. Quantitatively, the percentage of MitoSOX Red-positive cells (Figure 1d) and the fold change in median fluorescence intensity (MFI; Figure 1e) are significantly increased in the MGO MV-enriched fraction and MGO groups compared to Neg. CTL and CTL MV-enriched fraction controls, confirming robust mitochondrial ROS elevation following both MGO MV-enriched fraction treatment and MGO exposure. Notably, the MGO-derived MV-enriched fraction elicits a mitROS response comparable in magnitude to that induced by direct MGO exposure. Together, these results indicate that MGO-induced endothelial-derived MV-enriched fractions are potent mediators of mitROS production in recipient naïve endothelial cells, likely contributing to the propagation of oxidative stress and vascular dysfunction.

**Figure 2:**
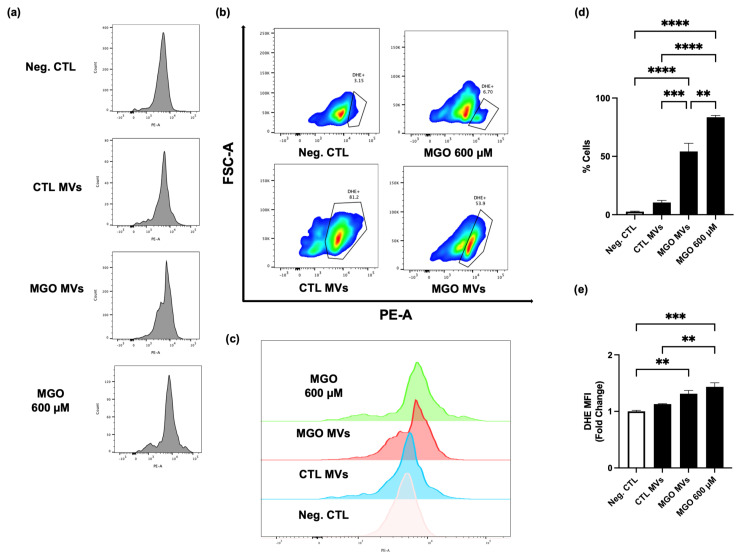
Intracellular Superoxide Analysis by Flow Cytometry Using Dihydroethidium (DHE) in Live EA.hy926 Cells. Flow cytometry analysis of intracellular superoxide levels in live EA.hy926 cells using DHE. Cells were left untreated, treated with control MV-enriched fraction or MGO-induced MV-enriched fraction (20 µg/mL, 24 h), or incubated with MGO (positive control; 600 µM, 18 h). (**a**) Representative histograms of DHE fluorescence at 24 h. The Y-axis represents the number of cells emitting fluorescence in the PE spectral region, while the X-axis represents the average fluorescence intensity. (**b**) Representative pseudocolor plots illustrating fluorescence distribution across experimental groups; the X-axis represents DHE fluorescence detected in the PE channel, and the Y-axis represents forward scatter area (FSC-A). (**c**) Half-offset histograms comparing fluorescence intensity distributions among all groups. (**d**) Bar graph showing the percentage of DHE-positive cells (mean ± SEM) for each condition. (**e**) Bar graph depicting the fold change in median fluorescence intensity (MFI) (mean ± SEM) across different treatments. All analyses were performed on at least three independent biological replicates; where each replicate represents independently treated endothelial cell culture wells/monolayers. ** *p* < 0.01, *** *p* < 0.001, and **** *p* < 0.0001 vs. Neg. CTL or indicated groups.

Moreover, analysis of total intracellular ROS (totROS) using DHE-based flow cytometry analysis (Figure 2) closely mirrors these findings. Representative DHE fluorescence histograms (Figure 2a), pseudocolor plots (Figure 2b), and comparative histograms (Figure 2c) collectively demonstrate increased fluorescence signals in both the MGO MV-enriched fraction and the MGO-treated groups compared with controls, indicative of enhanced intracellular superoxide formation. Bar graphs quantifying the percentage of DHE-positive cells (Figure 2d) and fold change in MFI (Figure 2e) confirm significant elevations under these conditions.

These complementary flow cytometry experiments underscore that the MGO-induced MV-enriched fraction not only boosts mitochondrial but also general intracellular superoxide levels, further supporting the role of MV-mediated ROS amplification as a mechanistic link between metabolic stress and endothelial dysfunction.

### 2.2. MGO-Induced Endothelial-Derived MV-Enriched Fraction Elevates ROS Levels in SESN2-Knockdown Endothelial Cells

**Figure 3:**
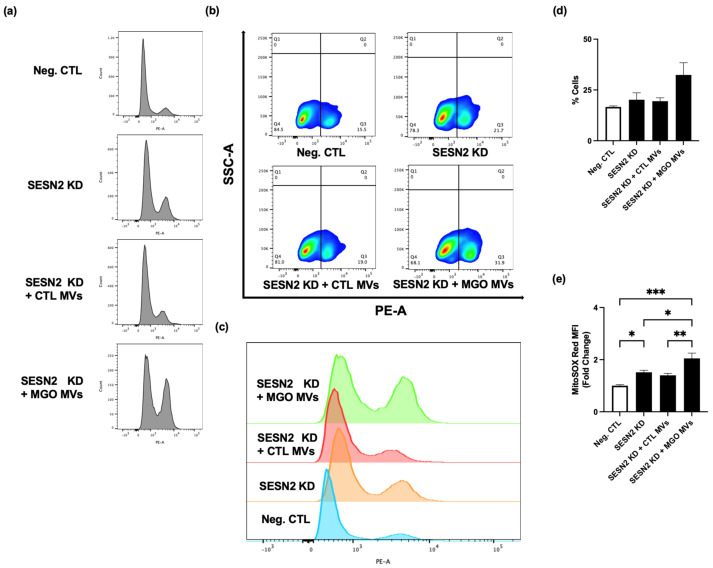
Mitochondrial Superoxide Analysis by Flow Cytometry Using MitoSOX Red in Live EA.hy926 Cells. Flow cytometry analysis of mitochondrial superoxide levels in live EA.hy926 cells using MitoSOX Red. Experimental conditions included untreated cells (Neg. CTL), SESN2 knockdown (SESN2 KD) cells, and SESN2 KD cells treated with either control MV-enriched fraction (CTL MV-enriched fraction) or MGO-induced MV-enriched fraction (MGO MV-enriched fraction) (20 µg/mL, 24 h). (**a**) Representative histograms of MitoSOX Red fluorescence at 24 h. The Y-axis represents the number of cells emitting fluorescence in the PE spectral region, while the X-axis represents the average fluorescence intensity. (**b**) Representative pseudocolor plots illustrating fluorescence distribution across experimental groups; the X-axis represents DHE fluorescence detected in the PE channel, and the Y-axis represents side scatter area (SSC-A). (**c**) Half-offset histogram comparing fluorescence intensity distributions among all groups. (**d**) Bar graph showing the percentage of MitoSOX Red-positive cells (mean ± SEM) for each condition. (**e**) Bar graph depicting the fold change in median fluorescence intensity (MFI) (mean ± SEM) across different treatments. All analyses were performed on at least three independent biological replicates; where each replicate represents independently treated endothelial cell culture wells/monolayers. * *p* < 0.05, ** *p* < 0.01, and *** *p* < 0.001 vs. Neg. CTL or indicated groups.

In addition to assessing ROS levels in naïve endothelial cells, we examined the effect of SESN2 silencing on redox balance. SESN2 is a key regulator of cellular antioxidant defense, and its knockdown by siRNA led to a marked elevation of mitROS, as shown by increased MitoSOX Red fluorescence compared to Neg. CTL (Figure 3). Exposing SESN2-knockdown cells to the control MV-enriched fraction resulted in a minor, statistically nonsignificant reduction in mitROS. By contrast, the incubation of cells with the MGO-induced MV-enriched fraction further exacerbated mitochondrial superoxide accumulation, significantly increasing both the percentage of MitoSOX Red-positive cells and the MFI compared to all other groups (Figure 3c–e).

**Figure 4:**
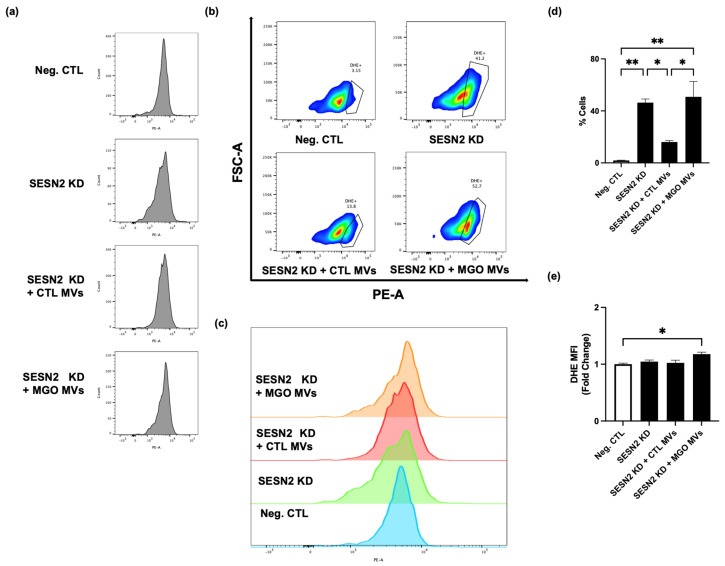
Intracellular Superoxide Analysis by Flow Cytometry Using Dihydroethidium (DHE) in Live EA.hy926 Cells. Flow cytometry analysis of intracellular superoxide levels in live EA.hy926 cells using DHE. Experimental conditions included untreated cells (Neg. CTL), SESN2 knockdown (SESN2 KD) cells, and SESN2 KD cells treated with either control MV-enriched fraction (CTL MV-enriched fraction) or MGO-induced MV-enriched fraction (MGO MV-enriched fraction) (20 µg/mL, 24 h). (**a**) Representative histograms of DHE fluorescence at 24 h. The Y-axis represents the number of cells emitting fluorescence in the PE spectral region, while the X-axis represents the average fluorescence intensity. (**b**) Representative pseudocolor plots illustrating fluorescence distribution across experimental groups; the X-axis represents DHE fluorescence detected in the PE channel, and the Y-axis represents forward scatter area (FSC-A). (**c**) Half-offset histogram comparing fluorescence intensity distributions among all groups. (**d**) Bar graph showing the percentage of DHE-positive cells (mean ± SEM) for each condition. (**e**) Bar graph depicting the fold change in median fluorescence intensity (MFI) (mean ± SEM) across different treatments. All analyses were performed on at least three independent biological replicates; where each replicate represents independently treated endothelial cell culture wells/monolayers. * *p* < 0.05, and ** *p* < 0.01 vs. Neg. CTL or indicated groups.

The evaluation of totROS using DHE staining showed that SESN2 silencing alone substantially increased ROS production compared to control cells (Figure 4). The MV-enriched fraction partially but significantly mitigated this oxidative stress, suggesting a cytoprotective effect. However, the MGO-induced MV-enriched fraction failed to significantly alter totROS levels compared with unchallenged SESN2-silenced cells (Figure 4).

Taken together, these findings demonstrate that SESN2 deficiency increases endothelial cell sensitivity to oxidative stress, with a pronounced effect on mitochondrial superoxide production. Importantly, the MGO-induced endothelial-derived MV-enriched fraction further amplifies mitROS but not totROS in SESN2-deficient cells, highlighting a selective exacerbation of mitochondrial redox imbalance. These findings are consistent with prior observations of increased mitROS in SESN2-silenced cells [16].

### 2.3. SESN2 Knockdown in Endothelial Cells Reduces NO with an Additive Reduction in MV-Enriched Fraction-Treated Cells

**Figure 5:**
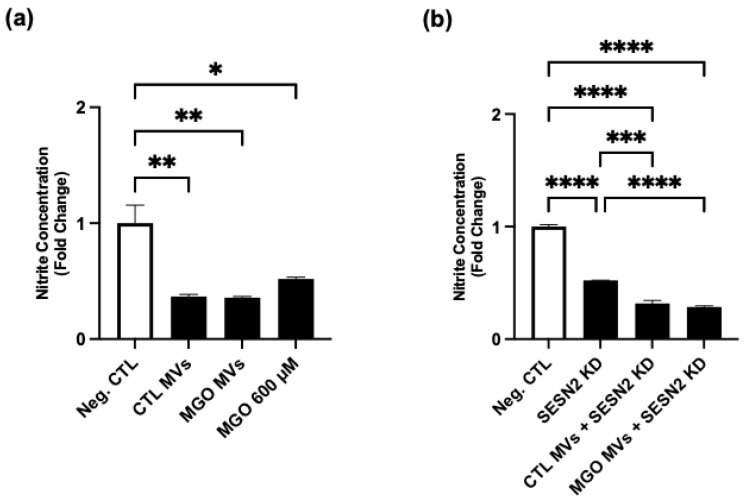
Nitrite production was assessed in naïve and SESN2 KD EA.hy926 endothelial cells. Cells were left untreated (Negative Control, Neg. CTL), treated with control MV-enriched fraction (CTL MV-enriched fraction) or MGO-induced MV-enriched fraction (MGO MV-enriched fraction) (20 µg/mL, 24 h), or incubated with MGO (600 µM, 18 h). Nitrite levels were measured using the 2,3-DAN assay. (**a**) Bar graph showing nitrite levels in SESN2-proficient cells. (**b**) Bar graph showing nitrite levels in SESN2 KD cells. Data are presented as mean ± SEM from three independent biological replicates; where each replicate represents independently treated endothelial cell culture wells/monolayers. * *p* < 0.05, ** *p* < 0.01, *** *p* < 0.001, and **** *p* < 0.0001 vs. Neg. CTL or indicated groups.

Following the evaluation of mitROS and totROS levels in normal and SESN2-deficient EA.hy926 cells challenged or not with the control or MGO-induced MV-enriched fraction, we investigated the impact of SESN2 deficiency on NO release in the presence or absence of exposure to the MV-enriched fraction. NO bioavailability, measured indirectly through nitrite production, was assessed in EA.hy926 endothelial cells that are proficient or deficient in SESN2 (KD). In SESN2-proficient cells, treatment with MGO (600 µM, 18 h) or either type of MV-enriched fraction (20 µg/mL, 24 h) led to a significant reduction in nitrite levels compared to untreated negative controls (Neg. CTL) (Figure 5a). SESN2-deficient cells displayed markedly reduced basal nitrite production in comparison to SESN2-proficient control cells (Figure 5b). Importantly, SESN2-KD cells exposed to the MGO MV-enriched fraction showed significantly lower nitrite levels than KD alone, indicating an additive inhibitory effect. Collectively, the data demonstrate that both SESN2 deficiency and MGO MV-enriched fraction treatment independently suppress endothelial NO bioavailability, and that their combination exerts a synergistic negative impact.

### 2.4. MGO-Induced Endothelial-Derived MV-Enriched Fraction Upregulates the Expression Profile of Non-Enzymatic and Enzymatic Antioxidants in Naïve Endothelial Cells

**Figure 6:**
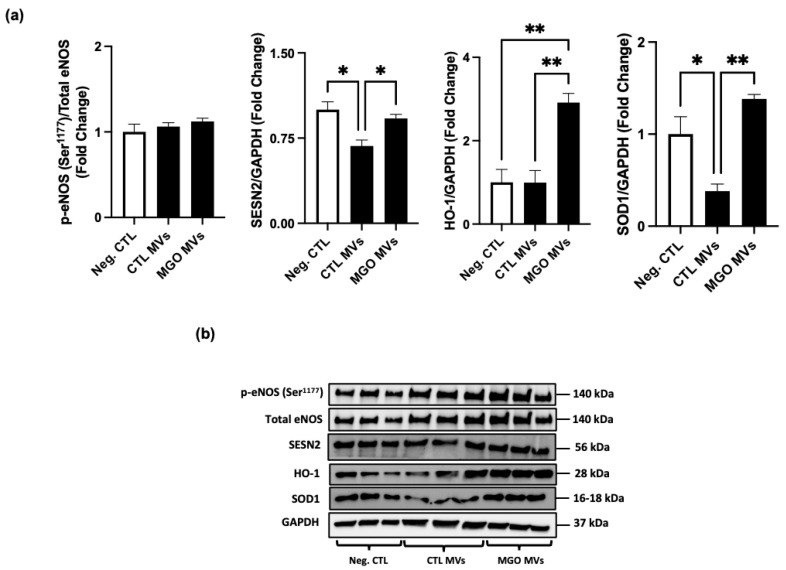
Effect of Endothelial-Derived MV-enriched fraction on the Protein Expression of Antioxidants in Naïve Endothelial Cells. EA.hy926 endothelial cells were left untreated (Neg. CTL) or incubated with either control MV-enriched fraction (CTL MV-enriched fraction) or MGO-induced MV-enriched fraction (MGO MV-enriched fraction) (20 µg/mL, 24 h). (**a**) Bars show densitometric analysis of protein expression levels of p-eNOS (Ser1177), total eNOS, SESN2, HO-1, and SOD1, normalized to the loading control GAPDH and expressed as a percentage of the untreated group (Neg. CTL). (**b**) Western blots of the investigated targets. Data are presented as mean ± SEM from three independent biological replicates; where each replicate represents independently treated endothelial cell culture wells/monolayers. * *p* < 0.05, and ** *p* < 0.01 vs. Neg. CTL or indicated groups.

To further investigate the cellular response to intercellular signals conveyed by the MV-enriched fraction from stressed, diabetes-mimicking cells, we examined the expression of antioxidant and endothelial function-related proteins in naïve EA.hy926 endothelial cells. A main objective of this study was to evaluate the protective role of SESN2 against endothelial dysfunction in a diabetic milieu. Exposure to the MGO-induced endothelial-derived MV-enriched fraction (20 µg/mL, 24 h) significantly upregulated SESN2 protein expression compared to cells treated with the control MV-enriched fraction (CTL MV-enriched fraction) (Figure 6). At the same time, the MGO-induced MV-enriched fraction enhanced the expression of the antioxidant enzymes SOD1 and HO-1, as confirmed by Western blotting and densitometric quantification (Figure 6). This antioxidant upregulation suggests that healthy endothelial cells activate compensatory defense mechanisms in response to potentially harmful signals transmitted by MGO-stressed cells via the shedding of the MV-enriched fraction.

In contrast, neither total eNOS nor its phosphorylated, activated form at Ser1177 showed significant alterations after incubation with either the control or MGO-induced MV-enriched fraction (Figure 6). This finding indicates that, while the MGO-induced MV-enriched fraction selectively enhances antioxidant defense pathways, it did not affect eNOS signaling under these conditions [21]. Altogether, these findings demonstrate that the MGO-induced MV-enriched fraction elicits a robust antioxidant protein response via the expression of SESN2, SOD1, and HO-1 in naïve endothelial cells, reflecting a potential adaptive or compensatory mechanism to counteract oxidative stress, while leaving eNOS pathways unaffected.

### 2.5. SESN2-Deficient Endothelial Cells Treated with MV-Enriched Fraction Derived from a Healthy Endothelium Mitigate the Antioxidant Response

**Figure 7:**
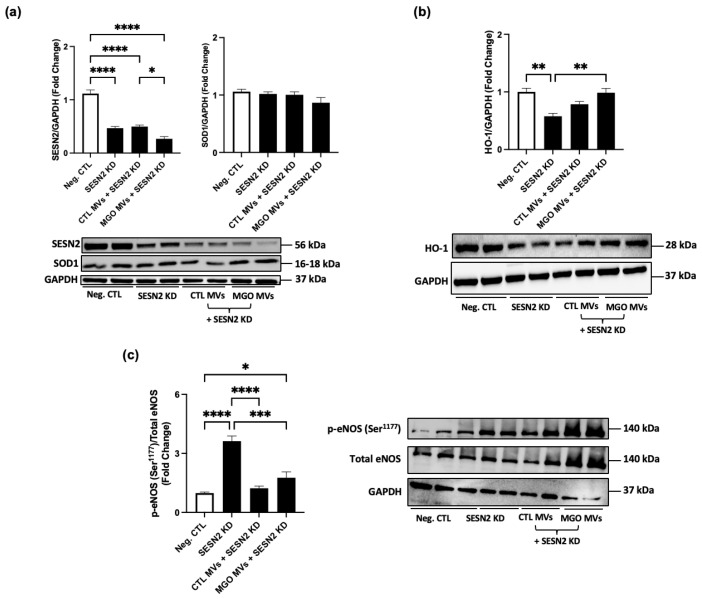
Effect of Endothelial-Derived MV-enriched fraction on the Protein Expression of Antioxidants in SESN2 Knockdown (KD) Endothelial Cells. SESN2 KD endothelial cells were left untreated (Neg. CTL) or incubated with either control MV-enriched fraction (CTL MV-enriched fraction) or MGO-induced MV-enriched fraction (MGO MV-enriched fraction) (20 µg/mL, 24 h). Western blot analysis and densitometric quantification of protein expression of (**a**) SESN2 & SOD1, (**b**) HO-1, and (**c**) p-eNOS (Ser1177) & total eNOS. All protein levels were normalized to the loading control GAPDH and expressed as a percentage of the untreated group (Neg. CTL). Data are presented as mean ± SEM (*n* = 3–6 per group) from three independent biological replicates; where each replicate represents independently treated endothelial cell culture wells/monolayers. * *p* < 0.05, ** *p* < 0.01, *** *p* < 0.001, **** *p* < 0.0001 vs. Neg. CTL or indicated groups.

We then investigated how SESN2 deficiency alters the expression of antioxidant and endothelial function-related proteins in response to the control or MGO-induced MV-enriched fraction. Protein levels were assessed by Western blot analysis and densitometric quantification (Figure 7). SESN2 KD cells displayed markedly reduced SESN2 protein expression compared to controls (Figure 7a), confirming effective knockdown. MGO-induced MV-enriched fractions significantly reduced SESN2 expression compared with CTL MV-enriched fractions in SESN2 KD cells. However, for SOD1, expression was not affected by either knockdown or MV-fraction treatment (Figure 7a). HO-1 expression was significantly reduced in SESN2-deficient cells (Figure 7b), consistent with the role of SESN2 in activating the Nrf2-dependent antioxidant pathway [22]. Interestingly, treatment of cells with the MGO-induced MV-enriched fraction significantly increased HO-1 levels, restoring expression to levels comparable to those in untreated control cells (Neg. CTL) (Figure 7b), thus maintaining a response similar to that of naïve endothelial cells. Phosphorylation of eNOS at Ser1177 was notably elevated in SESN2-deficient cells compared to controls (Figure 7c), consistent with prior evidence linking SESN2 to eNOS regulation [23]. Both the control and MGO-induced MV-enriched fraction significantly reduced the hyperphosphorylation of eNOS (Figure 7c).

In summary, SESN2 deficiency impaired basal antioxidant defenses, reduced SOD1 and HO-1, and enhanced eNOS phosphorylation at Ser1177. The control MV-enriched fraction partially restored SESN2 and SOD1 expression, whereas the MGO-induced MV-enriched fraction selectively upregulated HO-1 but did not rescue SESN2 levels. Both types of MV-enriched fraction attenuated the elevated eNOS phosphorylation observed in SESN2-deficient cells. These findings suggest that the MV-enriched fraction differentially modulates antioxidant responses and eNOS activation in the context of SESN2 deficiency.

## 3. Discussion

This study investigated the role of the MV-enriched fraction induced by MGO, an AGE precursor, in the endothelial oxidoreductase defense system and its potential to mediate or mitigate endothelial dysfunction. Numerous pathophysiological cellular pathways of diabetes and its complications have been explored through the years; endothelial dysfunction is an intersecting mechanism, also considered a ‘hub pathway’, connecting these disease-causing cellular changes [24]. Our recent work identified SESN2 as a central player contributing significantly to the intricate network orchestrating the pathology [14]. We established that *SESN2* loss induces a mesenchymal phenotypic transition in endothelial cells, which is indicative of endothelial dysfunction. However, the current study examined the condition from an alternative perspective, focusing on the role of the endothelial-derived MV-enriched fraction. Our findings indicated that the MGO-induced MV-enriched fraction markedly enhanced the expression of SESN2, SOD1, and HO-1 proteins in naïve endothelial cells compared to the control MV-enriched fraction. This indicates that the deleterious MV-enriched fraction transmits signals, presumably proteins and/or miRNAs, that stimulate these crucial redox regulators, allowing the cells to initiate a compensatory response to maintain redox equilibrium. This finding reinforces previous evidence that extracellular vesicles act as mediators of redox regulation [25,26]. The difference observed between our previous study and the current one can be explained simply by the distinction between a direct insult to cells and an indirect signal conveyed by stressed cells. In this scenario, healthy cells that receive signals from MGO-exposed cells face a less severe challenge than those with direct MGO exposure, thereby retaining a greater ability to initiate protective responses and avoid the same detrimental trajectory. Moreover, this study models the propagation of this signal across diverse vascular territories, such as the cardiac and renal vasculature, in a diabetes-relevant context. As for discrepancies between our findings and other studies using MGO, several factors may account for these differences. MGO treatment conditions vary considerably across studies. In our group’s work, we determined an MGO concentration of 600 µM following comprehensive optimization experiments to identify the concentration associated with reduced SESN2 expression, which reflects diabetes conditions observed in patients. This choice is supported by numerous studies showing that diabetic patients exhibit markedly reduced circulating SESN2 levels compared with healthy individuals [27,28,29,30].

Despite the upregulation of antioxidant proteins in treated endothelial cells, ROS levels continued to rise, driven by superoxide anion, the main contributor in the vasculature and the precursor of most other ROS species [29]. This may be because the persistent oxidative assault generates ROS at a pace that outstrips the cell’s current neutralization capabilities, despite the cell bolstering its antioxidant arsenal. A study by Jansen et al. showed that a high-glucose environment increases NADPH oxidase activity in endothelial microparticles, promoting inflammation and endothelial dysfunction, findings that agree with our results [30]. Furthermore, we found that the MV-enriched fraction, whether CTL- or MGO-induced, significantly reduced nitrite levels compared with non-treated naïve endothelial cells, indicating a negative impact on endothelial health. There are several potential reasons for both types of MV-enriched fraction having the same effect. First of all, the similar nitrite levels may indicate that both MV populations are biologically active and capable of modulating nitric oxide bioavailability in recipient endothelial cells. As nitrite represents the net balance between NO production and oxidative consumption, comparable levels across groups do not necessarily exclude mechanistic differences in endothelial responses. Secondly, as evident from ROS flow cytometry-based assays, the MGO MV-enriched fraction caused a significant increase in ROS compared to the CTL MV-enriched fraction, demonstrating distinct cargo and distinct effects on recipient cells. Thirdly, most assays developed to detect NO can do so only indirectly by measuring a stable metabolite, such as nitrite; direct measurement with methods such as electron paramagnetic resonance (EPR) would provide a clearer, more accurate representation [31].

In addition to the three antioxidants (SESN2, SOD1, and HO-1) examined, we also assessed eNOS, an indirect marker of endothelial health, as it is the primary enzyme responsible for NO production in the endothelium. Our results show that phosphorylation at the active site (Ser1177) of eNOS was not affected by treatment with the MV-enriched fraction, despite differences in nitrite levels between the treatment groups and the non-treated control group, and in ROS levels as well. This can be due to a self-amplifying feedback loop in which peroxynitrite (ONOO^−^), an RNS produced during oxidative stress, binds to eNOS, deactivating it and eventually disrupting NO production [32]. So, despite having a fairly basal level of ‘activated’ eNOS, the NO produced reacts with superoxide-producing peroxynitrite, which causes endothelial damage and further ROS production, and hence, we fail to observe the corresponding nitrite levels that match the eNOS storyline between the different groups, as the NO is being consumed by its reaction with superoxide [33].

Another key focus of this study, beyond the role of the MV-enriched fraction, was to examine how SESN2 deficiency alters cellular responses to the MGO MV-enriched fraction. Interestingly, SESN2 expression showed an opposite trend to earlier observations, with the control MV-enriched fraction inducing higher SESN2 levels than the MGO MV-enriched fraction. This suggests that the combined stress of the MGO MV-enriched fraction and *SESN2* knockdown exceeds the cells’ capacity to mount an effective protective response. Consistent with this, SOD1 and HO-1 did not differ between MV types in SESN2-deficient cells, likely reflecting SESN2’s regulatory role over these antioxidant pathways. In contrast, ROS production was markedly higher in response to the MGO MV-enriched fraction compared to the control MV-enriched fraction under SESN2-deficient conditions. Together, these findings indicate that the combined insult places greater stress on endothelial cells than either condition alone, reinforcing the critical role SESN2 plays in endothelial dysfunction in hyperglycemia.

A key and novel finding of our study is the link between SESN2 and eNOS activity. We showed for the first time that SESN2 deficiency leads to a significant increase in eNOS phosphorylation at Ser1177 (activatory site). However, it is important to interpret such results with caution, as eNOS phosphorylation does not always equate to dimerization (i.e., coupling) of the enzyme, which is essential for its activity [34]. To further validate or contest this finding, it was essential to evaluate the functional outcome of eNOS activity, specifically, NO generation. To this end, we quantified nitrite, a stable surrogate metabolite of NO. Our findings indicated that SESN2-deficient endothelial cells had markedly lower nitrite levels than untreated (naïve) cells. This indicates that eNOS was probably uncoupled and, despite phosphorylation at Ser1177, was unable to produce adequate NO. Another possibility is that any nitric oxide generated quickly interacts with superoxide to form peroxynitrite, thereby exacerbating oxidative stress in these cells.

Several limitations should be acknowledged. First, this study used EA.hy926 endothelial cells, an immortalized hybrid endothelial cell line. Although this model is widely used for mechanistic studies of endothelial function, oxidative stress, and inflammatory signaling, it may not fully reproduce the phenotype, heterogeneity, or vascular-bed-specific responses of primary endothelial cells. Therefore, the present findings should be interpreted as evidence from a controlled in vitro endothelial model and require validation in primary endothelial cells, such as HUVECs or microvascular endothelial cells, as well as in more physiologically relevant in vivo or patient-derived models.

Second, the isolated vesicle preparation was defined operationally as an MV-enriched or large EV-enriched fraction obtained by differential centrifugation at 21,000× *g*. Comprehensive vesicle characterization by particle-size analysis, electron microscopy, and EV marker profiling was not performed; therefore, the presence of non-vesicular material or other large extracellular particles cannot be fully excluded. Accordingly, the biological effects observed in this study are attributed to the isolated MV-enriched fraction rather than to purified MVs alone.

Third, ROS detection was based on MitoSOX Red and DHE fluorescence, which are superoxide-sensitive probes. Other ROS species were not directly measured. Therefore, the data should be interpreted as evidence of increased mitochondrial and intracellular superoxide-associated oxidative stress rather than a complete profile of all reactive oxygen species. Finally, AMPK/Nrf2 signaling, eNOS coupling status, and direct NO or peroxynitrite measurements were not assessed, limiting mechanistic conclusions regarding the pathways linking SESN2 to antioxidant responses and NO bioavailability.

This study offers significant insights into the interplay between the diabetic MV-enriched fraction, SESN2, and redox imbalance in endothelial dysfunction. Our study significantly extends the existing evidence linking the MV-enriched fraction to redox signaling proteins in the context of diabetes-induced endothelial dysfunction. While prior studies have predominantly focused on the role of the MV-enriched fraction in the development and complications of diabetes via inflammatory pathways, our findings underscore its particular relevance in regulating redox signaling in response to the metabolic stressor MGO. Moreover, this work establishes a novel link between SESN2 and eNOS, paving the way for further exploration of their interplay in diabetic vascular pathophysiology. Expanding on our findings in subsequent research can enhance our comprehension of how diabetic MV-enriched fractions modulate redox signaling, influence endothelial function, and serve as potential therapeutic targets in diabetic vascular disease.

## 4. Materials and Methods

### 4.1. Cell Culture

EA.hy926 (ATCC^®^ CRL-2922™, ATCC, Manassas, VA, USA) endothelial cells were cultured in high-glucose Dulbecco’s Modified Eagle’s Medium (DMEM) supplemented with fetal bovine serum (FBS, 10%), penicillin/streptomycin (1%), sodium pyruvate (1%), and L-glutamine (1%) from Gibco (Thermo Fisher Scientific, Waltham, MA, USA). Cells were left to adhere and proliferate at 37 °C, 5% carbon dioxide (CO_2_), and 95% humidity within an incubator. Cells used in experimental studies ranged from passage 5 to 15.

### 4.2. Cell Treatments

All treatments were performed in 6-well plates, with each well seeded with 200,000 endothelial cells. After a 24-h adhesion period, cells were treated with methylglyoxal (MGO) at 600 μM for 18 h to simulate diabetic conditions (Sigma-Aldrich, Darmstadt, Germany). MGO serves as a precursor to advanced glycation end-products (AGEs) [14]. This particular concentration was chosen based on our prior publications [14,35]. It consistently produced endothelial redox impairment while preserving adequate cell viability for subsequent functional and molecular analyses.

For MV-enriched fraction treatment of endothelial cells, a concentration of 20 µg/mL was used for 24 h. This concentration was selected based on previous extracellular vesicle treatment studies [36,37] and internal optimization, which showed that this dose is sufficient to induce measurable biological responses in recipient endothelial cells.

### 4.3. Isolation of Microvesicles (MV-Enriched Fraction)

To minimize contamination from serum-derived vesicles, fetal bovine serum (FBS) was first subjected to ultracentrifugation at 52,000× *g* for 2 h using a Sorvall LYNX 4000 Superspeed Centrifuge (Thermo Scientific, Waltham, MA, USA). The MV-enriched fraction was isolated from the conditioned medium of treated and non-treated endothelial cells. After treating the cells with MGO prepared in the FBS-depleted medium for 18 h, the media were collected in Nalgene™ Oak Ridge High-Speed PPCO Centrifuge Tubes (Thermo Scientific, Waltham, MA, USA) and centrifuged at 21,000× *g* for 30 min. The supernatant from the first centrifugation was kept for nitrite measurement as described below, while the pellet was washed with phosphate-buffered saline (PBS; Thermo Fisher Scientific, Waltham, MA, USA) for two more rounds of equal-speed centrifugations AKA ‘differential centrifugation’, which is a commonly used and established method for the isolation of the different EVs subtypes depending on the centrifugal force used [38].

### 4.4. Experimental Groups

The experimental design included 3 groups: (1) untreated control (Neg. CTL): EA.hy926 cells cultured in MV-depleted DMEM only, (2) control MV-enriched fraction (CTL MV-enriched fraction) treatment: EA.hy926 cells treated with the MV-enriched fraction isolated from non-treated cells, and (3) MGO MV-enriched fraction treatment: EA.hy926 cells treated with the MV-enriched fraction isolated from MGO-treated cells.

### 4.5. Flow Cytometric Analysis of Intracellular Reactive Oxygen Species (ROS)

Dihydroethidium (DHE), a fluorescent probe (Thermo Scientific, Waltham, MA, USA), was used to detect superoxide anion (O_2_^−^) in endothelial cells. First, cells were seeded in 6-well plates. Cells were trypsinized and collected. Harvested cells were incubated with 10 μM DHE for 30 min at 37 °C in the dark. Red fluorescence was measured by flow cytometry using the PE channel on a FACSAria III (BD Biosciences, San Jose, CA, USA). Flow cytometry analysis was conducted in duplicate in at least three independent biological experiments. A separate live/dead viability dye was not used in these experiments. Instead, analysis was restricted to the main intact cell population based on forward- and side-scatter properties, and debris was excluded prior to fluorescence analysis. Doublets were excluded using standard FSC-A/FSC-H gating. ROS-positive cells were then quantified from the gated intact-cell population using DHE fluorescence intensity in the PE channel. MGO was used as a positive control.

### 4.6. Flow Cytometric Analysis of Mitochondrial ROS

A MitoSOX™ Mitochondrial Superoxide Red fluorescent probe (Thermo Scientific, Waltham, MA, USA) was used for detecting superoxide anion (O_2_^−^) in the mitochondria of endothelial cells. First, cells were seeded in 6-well plates. Cells were trypsinized and collected. Harvested cells were incubated with 5 μM MitoSOX for 30 min at 37 °C in the dark. Red fluorescence was measured by flow cytometry using the PE channel on a FACSAria III. Flow cytometry analysis was conducted in duplicate in at least three independent biological experiments. A separate live/dead viability dye was not used in these experiments. Instead, analysis was restricted to the main intact cell population based on forward- and side-scatter properties, and debris was excluded prior to fluorescence analysis. Doublets were excluded using standard FSC-A/FSC-H gating. ROS-positive cells were then quantified from the gated intact-cell population using MitoSOX Red fluorescence intensity in the PE channel. MGO was used as a positive control.

### 4.7. Nitrite Detection Assay

2,3-Diaminonaphthalene (DAN) (Combi-blocks, San Diego, CA, USA) was used to detect nitrite concentrations in the conditioned medium of treated cells. DAN was prepared in 0.62 M HCl to a final concentration of 0.31 mM. First, phenol red-free conditioned cell culture media were collected in microcentrifuge tubes. Then, an equal volume of chilled acetonitrile was added to the samples, vortexed, and centrifuged at approximately 12,000× *g* for 10 min to precipitate/remove large molecular weight proteins that can interfere with the assay’s efficacy [39]. A standard curve was generated using serial dilutions of sodium nitrite (0–200 µM). In a black-walled 96-well plate, 100 µL of each standard or sample was mixed with an equal volume of double-distilled water and 20 µL of the prepared DAN solution. The mixture was incubated in the dark at room temperature for 15 min. After incubation, 10 μL of 2.8 M NaOH was added to halt the reaction and establish alkaline conditions for optimal fluorescence. Fluorescence measurements were recorded using a BioTek Synergy H1 microplate reader (Agilent Technologies, Winooski, VT, USA) with Gen5 version 2.0 software, at an excitation wavelength of 370 nm and an emission wavelength of 450 nm.

### 4.8. Western Blot Analysis

After treatment, the culture plates were transferred to ice. The culture medium was carefully aspirated and discarded, and the cells were rinsed twice with ice-cold PBS to remove any residual medium. For protein extraction, ice-cold radioimmunoprecipitation assay (RIPA) buffer containing Tris (0.5 M, pH 6.8) and sodium dodecyl sulfate (SDS, 20%; Thermo Fisher Scientific) was freshly supplemented with a protease and phosphatase inhibitor cocktail tablet (Thermo Fisher Scientific). The resulting protein lysates were collected for subsequent analyses. Protein concentration was measured using the Pierce™ bicinchoninic acid (BCA) protein assay (Thermo Fisher Scientific). Equal amounts of protein lysate (15–25 µg) were resolved on SDS-PAGE gels ranging from 8–12%, depending on the molecular weight of the target proteins, and then transferred onto PVDF membranes (Thermo Fisher Scientific, Waltham, MA, USA). Membranes were blocked for one hour at room temperature in tris-buffered saline containing 0.1% Tween 20 (TBS-T; Sigma-Aldrich, Hamburg, Germany) supplemented with 5% skimmed milk. After blocking, membranes were washed with TBS-T and incubated overnight at 4 °C on a shaker with the following primary antibodies: SESN2 (#8487S), HO-1 (#43966S), SOD1 (#37385S), phospho-eNOS (Ser1177) (C9C3) (#9570S), eNOS (D9A5L) (#32027S), and GAPDH (#2118S) (Cell Signaling Technology, Danvers, MA, USA). On the following day, membranes were washed with TBS-T and incubated for one hour at room temperature with horseradish peroxidase (HRP)-conjugated secondary antibodies, either anti-rabbit (#7074S) or anti-mouse (#7076S; Cell Signaling Technology), diluted at 1:3000. Protein bands were detected using the ECL Substrate Kit (High Sensitivity) (#ab133406; Abcam, Cambridge, UK) and imaged with the Bio-Rad ChemiDoc MP imaging system (Bio-Rad, Hercules, CA, USA). Densitometric quantification of protein bands was performed using Image Lab Software for Mac, version 6.1 (Bio-Rad, Hercules, CA, USA).

### 4.9. SESN2 Knockdown via siRNA

*SESN2* and control siRNA duplexes were designed and synthesized by Integrated DNA Technologies (IDT, Coralville, IA, USA) for *SESN2* gene silencing assays. The siRNA sequence targeting SESN2 was designed as follows: sense (5′-CUA GUU CAG UUU UUU GAC); anti-sense (5′-AAA GGA AGU CAA AAA ACU). Briefly, 200,000 endothelial cells were seeded onto 6-well plates and allowed to adhere for 24 h. Then, cells were transfected with *SESN2* siRNA duplexes according to the manufacturer’s protocol. For transfection preparation, 200 μL of serum-free DMEM (Thermo Scientific, Waltham, MA, USA) was dispensed into a microcentrifuge tube. siRNA duplexes were solubilized in the added media, followed by the addition of INTERFERin^®^ transfection reagent (Polyplus, Illkirch, France). The mixture was then properly vortexed and incubated at room temperature for 10 min. After incubation, the transfection mixture was added to each well containing 2 mL of fresh complete medium. The cells were subsequently incubated for an additional 24 h, resulting in a total incubation period of 48 h post-transfection to allow for *SESN2* silencing. The *SESN2*-silenced cells were treated with the MV-enriched fraction 24 h after transfection.

### 4.10. Statistical Analysis

Results are presented as mean ± SEM, with *n* being the number of biological replicates. The statistical analyses were conducted using GraphPad Prism^®^ 10.4.1 software for Mac. The Shapiro–Wilk normality test was used to assess data normality on each occasion. A one-way ANOVA, accompanied by Tukey’s multiple comparisons post hoc test, was utilized for group comparison of data exhibiting a Gaussian distribution. The Kruskal–Wallis test, along with Dunn’s multiple comparisons post hoc test, was employed for group comparison of non-Gaussian data. Statistical significance was established as a two-tailed *p* value of <0.05.

## Data Availability

The original contributions presented in this study are included in the article. Further inquiries can be directed to the corresponding author.

## Abbreviations

The following abbreviations are used in this manuscript:

AGE	Advanced glycation end-product
AGEs	Advanced glycation end-products
AMPK	AMP-activated protein kinase
CTL	Control
CVDs	Cardiovascular diseases
DAN	2,3-diaminonaphthalene
DHE	Dihydroethidium
eNOS	Endothelial nitric oxide synthase
EPR	Electron paramagnetic resonance
ER	Endoplasmic reticulum
EVs	Extracellular vesicles
FBS	Fetal bovine serum
HO-1	Heme oxygenase-1
MGO	Methylglyoxal
mitROS	Mitochondrial reactive oxygen species
MVs	Microvesicles
NADPH	Nicotinamide adenine dinucleotide phosphate
NO	Nitric oxide
Nrf2	Nuclear factor erythroid 2-related factor 2
RAGE	Receptor for advanced glycation end-products
ROS	Reactive oxygen species
SEM	Standard error of the mean
SESN1	Sestrin 1
SESN2	Sestrin 2
SESN3	Sestrin 3
siRNA	Small interfering RNA
SOD1	Superoxide dismutase 1
SODs	Superoxide dismutases
T2DM	Type 2 diabetes mellitus
totROS	Total reactive oxygen species
